# Recombinant proteins A29L, M1R, A35R, and B6R vaccination protects mice from mpox virus challenge

**DOI:** 10.3389/fimmu.2023.1203410

**Published:** 2023-06-26

**Authors:** Ding Tang, Xiaoke Liu, Jia Lu, Huifen Fan, Xiuli Xu, Kaili Sun, Ruyu Wang, Chunyang Li, Demiao Dan, Hongqiao Du, Zejun Wang, Xinguo Li, Xiaoming Yang

**Affiliations:** Biosafety Level 3 Laboratory, Wuhan Institute of Biological Products Co., Ltd., Wuhan, China

**Keywords:** mpox virus, recombinant protein, vaccination, antibody response, virus challenge

## Abstract

Since May 2022, mutant strains of mpox (formerly monkeypox) virus (MPXV) have been rapidly spreading among individuals who have not traveled to endemic areas in multiple locations, including Europe and the United States. Both intracellular and extracellular forms of mpox virus have multiple outer membrane proteins that can stimulate immune response. Here, we investigated the immunogenicity of MPXV structural proteins such as A29L, M1R, A35R, and B6R as a combination vaccine, and the protective effect against the 2022 mpox mutant strain was also evaluated in BALB/c mice. After mixed 15 μg QS-21 adjuvant, all four virus structural proteins were administered subcutaneously to mice. Antibody titers in mouse sera rose sharply after the initial boost, along with an increased capacity of immune cells to produce IFN-γ alongside an elevated level of cellular immunity mediated by Th1 cells. The vaccine-induced neutralizing antibodies significantly inhibited the replication of MPXV in mice and reduced the pathological damage of organs. This study demonstrates the feasibility of a multiple recombinant vaccine for MPXV variant strains.

## 1 Introduction

Mpox virus (MPXV) belongs to the *Orthopoxvirus* genus in the family of *Poxviridae* and consists of the following two clades: the West and Central African clades (1, 2). The strain prevalent in the 2022 mpox epidemic originated from the West African branch, which was less lethal relative to the Central African branch, although produced higher mutation rate than predicted. The 2022 MPXV diverged from the related 2018–2019 viruses by a mean of 50 single-nucleotide polymorphisms (SNPs), which is far more than expected considering previous estimates of the substitution rate for *Orthopoxviruses (* 3). Due to the possible negative impact once mpox outbroke on human immunity, there has been a renewed interest in the availability of a safe and effective mpox vaccine, particularly against this new 2022 mutant strain. Although smallpox vaccines can offer some protection, widespread skin rashes and atopic dermatitis often occur as a side effect of the vaccination. In more severe cases, this can even lead to life-threatening side effects such as myocarditis and pericarditis (4, 5). Furthermore, attenuated vaccines may revert to pathogenic forms (6). Meanwhile, fears of smallpox virus being used as a biochemical weapon have given further impetus to the development of a safer mpox vaccine (7). Recombinant protein vaccines are prepared by purifying the expressed target protein into a vaccine that does not replicate in the body; this high level of safety is the basis for an alternative to the attenuated vaccines.

The rational design of a recombinant vaccine depends on the classical structure of the *Orthopoxvirus* and the immunogenicity of the selected protein. Similar to other poxviruses, MPXV also has two forms, namely, extracellular enveloped virus (EEV) and intracellular mature virus (IMV). The most abundant particles are the IMVs, which accumulate in infected cells and can be released as cells die. Meanwhile, EEVs have undergone exocytosis from cells at the plasma membrane to enhance spread within the host and are essentially the IMVs with additional membrane. However, they are highly susceptible to rupture, whereas the EEV membrane bear several important neutralizing epitopes (8, 9). The surface proteins of IMV and EEV are highly differentiated, and studies on smallpox virus have shown that a single protein does not induce as much immunogenicity as multiple proteins in combination (6, 10). Therefore, a mixture of four proteins was selected as a component of the recombinant vaccine in this study. A29L is a surface membrane fusion protein located on the IMV and is responsible for binding to cell surface heparin (11). Additionally, A35R is an envelope glycoprotein in EEV that plays a key role in the formation of effective EEVs, while contributing to the effective intercellular spread of viral particles and being required for the formation of actin-containing microvilli and intercellular spreading (12). Meanwhile, M1R is a highly conserved myristate surface membrane protein in IMV involved in viral particle assembling, and entry (13). Furthermore, B6R is a palmitoylated glycoprotein in EEV which is required for effective cell spreading, while also being involved in complement control (14, 15). All of these proteins or their homologues in smallpox virus and cowpox virus have been shown to be important neutralizing antibody targets (2, 16).

QS-21 is a water-soluble triterpene glycoside extracted from the bark of *Quillaja saponaria*, which has become the first choice adjuvant in many vaccine clinical trials (17). QS-21 functions as an immunostimulatory adjuvant, stimulating antibody-based and cell-mediated immune responses by acting on antigen presenting cells (APCs) and T cells to produce high titers of antibodies, which is significantly superior to other adjuvants, such as aluminum adjuvant and MF59, in terms of enhancing antibody and T-cell responses to target antigens (18, 19). In addition, it can stimulate the production of antigen-specific cytotoxic T lymphocytes, while inducing the production of Th1 cytokines, interleukin-2, and interferon-gamma (IL-2 and IFN-γ) (20–23). In terms of animal model selection, Ground squirrels, black-tailed prairie dogs, and African dormice are highly susceptible to MPXV (24–27). However, these animals are currently not being bred as experimental animals. In addition, the lack of specific immunological reagents targeting these animals can make the experiment complex and limited. BALB/c and C57BL/6 mice were found to be resistant to MPXV, however, some studies have found that a large number of viruses can still be detected in the ovary, lung, and spleen of BALB/c between 96-120 h after being challenged by the MPXV virus (28). After 240 h, the virus could not be detected, indicating that these animals had cleared the infection (29). Therefore, 96-120 h can be selected as the euthanasia time point for non-lethal models for vaccine evaluation.

In this study, BALB/c mice were immunized with recombinant A29L, M1R, A35R, and B6R proteins combined with QS-21 adjuvant. Sera were collected for neutralizing and binding antibodies test. Then mice were challenged with MPXV, and the viral loads and histopathological changes in organs were examined. Experimental results show that this vaccine induced strong antigen-specific humoral and cellular immune responses in mice. More importantly, it showed excellent protection in MPXV challenge. This information can contribute for further research on mpox vaccines.

## 2 Materials and methods

### 2.1 Viruses and cells

Vero E6 cells were maintained at 37 °C with 5% CO_2_ in Dulbecco’s modified Eagle’s Medium (DMEM, Gibco, New York, NY, USA) supplemented with 10% heat-inactivated fetal bovine serum (FBS, Every Green, Zhejiang, China) alongside 50 units/mL penicillin–streptomycin (Gibco, New York, NY, USA). Meanwhile, the MPXV strain (WIBP-MPXV-001) was cultured in Vero E6 cells. The confluent monolayers Vero E6 cells were infected at a multiplicity of infection (MOI) of 2 for 72 h at 37 °C, followed by three repeated freeze-thaws. Cell debris were then precipitated and removed by centrifugation at 4,000 × *g* for 5 min. Subsequently, the supernatant was harvested and stored at -80°C as the virulent strain for the MPXV attack challenge for this experiment. All virus culture and challenge were performed in a biosafety level 3 facility (Wuhan Institute of Biological Products Co., Ltd., Wuhan City, China).

### 2.2 Recombinant proteins and QS-21

Soluble forms of the MPXV recombinant proteins of A29L, M1R, A35R, and B6R (Vazyme, Nanjing, China) were marked with a His-tag respectively. QS-21 (MedChem Express, Monmouth Junction, NJ, USA) were purchased from MedChem Express and was used as vaccine adjuvant (30).

### 2.3 Western blotting

ACE2-293T cells transformed with pcDNA 3.1-A29L-His tag, pcDNA 3.1-B6R-His tag, pcDNA 3.1-A35R-His tag, and pcDNA 3.1-M1R-His tag were propagated and harvested, respectively. Cell from cultures were resuspended in Laemelli SDS sample buffer (Thermo Fisher Scientific, Waltham, MA, USA) and heated at 95°C for 10 min. These samples were separated on 12% SDS-PAGE, transferred to PVDF membrane (Sangon Biotech, Shanghai, China) and blocked with 5% skimmed milk-Tris buffered saline amended with 0.1% Tween 20 (TBS-T, Sangon Biotech, Shanghai, China) overnight at 4°C. Then wash three times, membrane was incubated for 1 h with anti-A29L, anti-B6R, anti-A35R and anti-M1R antibodies (Vazyme, Nanjing, China) at RT with continuous slow shaking, respectively. Membrane was incubated with 1:3000 anti-rabbit IgG conjugated to horseradish peroxidase (Sigma-Aldrich, St. Louis, MO, USA) for 30 min at RT. Signals were detected using SuperSignal™ West Femto Maximum Sensitivity Substrate (Thermo Fisher Scientific, Waltham, MA, USA).

### 2.4 Mouse immunization and challenge protocol

Here, 5-to-6-week-old female BALB/c mice were supplied by the animal reproduction facilities of the Wuhan Institute of Biological Products (WIBP) and subsequently kept in the animal laboratory of WIBP. All mice were randomly divided into three groups of eight mice each. A mixture of 15 μg each of A29L, M1R, A35R, and B6R was made and then this mixture was mixed with 15 μg of QS-21 in phosphate-buffered saline (PBS) to compose a recombinant protein vaccine. Mice in the vaccine group were injected subcutaneously with this recombinant protein vaccine on days 0, 21, and 42. Meanwhile, the mice in the QS-21 group were injected subcutaneously with 15 μg QS-21, whereas the mice in PBS group were subcutaneously injected with PBS.

Two weeks after the third immunization, three mice from each group were randomly selected to measured T cell responses, and the remain mice (n = 5 mice/group) were anesthetized with isopentane and inoculated intranasally and intraperitoneally using 20 μL and 480 µL of the MPXV, respectively. MPXV titer was 2.24 × 10 ^8^ PFU/mL. Additionally, the weight and clinical symptoms were monitored during the experiment.

### 2.5 Sample collection

Eight mice from each group had venous blood collected through the infraorbital plexus on days 0, 21, 42, and 56. Three mice from each group were randomly selected to be sacrificed and collected spleens on day 56. Five days after viral challenge, the remaining five mice from each group were euthanized and blood samples, spleens, lungs and ovaries were all collected. Blood was then incubated at 37°C for 1 h, before being maintained overnight at 4°C to shrink the clot, and subsequently centrifuged at a relative centrifugal force of 2,000 × g for 10 min. Sera were then collected and stored at -80°C until later use.

### 2.6 Immunofluorescence staining

Vero E6 cells were inoculated in 24-well plates the day before transfection, with transfection starting when cell density reached 80%. System A was 500 μL OPTI-MEM mixed with 10 μg plasmid pcDNA3.1-His-C (Fenghui Biotechnology Co., Ltd, Hunan, China) and then left at room temperature (RT) for 5 min. Meanwhile, System B was 500 μL OPTI-MEM mixed with 20 μL Lipofectamine 2000 (invitrogen, Carlsbad, CA, USA), and then left at RT for 5 min. Systems A and B were then mixed and allowed to stand for 15 min, before being inoculated into 24-well plates with 100 μL per well. The plates were incubated in the carbon-dioxide incubator at 37°C for 6 h, and the medium was changed to DMEM supplemented with 10% FBS and 50 units/mL penicillin–streptomycin and subsequently incubated for 60 h. Cells were washed with PBS and then incubated in PBS containing 5% BSA (YHSM, Beijing, China) and 1% Triton X-100 (Sinopharm Chemical Reagent Co., Ltd, Shanghai, China) at RT for 1 h, followed by incubation overnight at 4°C with primary antibodies. The primary antibody for the positive control was 6 × His Tag Monoclonal Antibody (invitrogen, Carlsbad, CA, USA), and for the rest of the group was mouse serum. After washing to remove primary antibody, cells were incubated with FITC-conjugated AffiniPure Goat Anti-mouse IgG(H+L) (Boster, Wuhan, China) at RT for 1 h, and then washed thoroughly. Cell nuclei were stained with DAPI (Solarbio, Beijing, China) for 10 min, followed by washing thoroughly again and being photographed under a fluorescent microscope to collect images.

### 2.7 Enzyme-linked immunosorbent assay

Polystyrene 96-well round bottom plates (Corning, New York, NY, USA) were coated with recombinant proteins A29L, M1R, A35R, and B6R in 0.05 M carbonate buffer (15 mM Na_2_CO_3_ and 3.5 mM NaHCO_3_) at 4°C overnight. Plates were then washed with PBS-0.05% Tween 20 and blocked with PBS-10% FBS at 37°C for 1 h. The serum was heat-inactivated at 56°C for 30 min prior to analysis. Two-fold serial dilutions of sera in PBS-10% FBS were then added to the blocked plates and incubated at 37°C for 1 h. After washing, horseradish peroxidase (HRP)-conjugated goat anti-mouse IgG antibody (diluted 1:20,000; BOSTER, Wuhan, China) in PBS-10% FBS was added to the wells and then incubated at 37°C for 1 h. After washing, ELISA was performed by adding a 1:1 mixture of tetramethylbenzidine and hydrogen peroxide. (Sangon Biotech, Shanghai, China) Color development was subsequently stopped with 2 N sulfuric acid, and the absorbance was measured at 450 nm on an Epoch Microplate Reader (Agilent Technologies, Santa Clara, CA, USA) with the reference filter set at 630 nm. There were 8 samples from each group, and three replicates were set up for each sample.

### 2.8 MPXV plaque reduction neutralization tests

Vero E6 cells were seeded 1 day prior to the experiment in 12-well plates (Corning, New York, NY, USA) at a density of 2 × 10^5^ cells per well. Serum samples were heated at 56 °C for 30 min to inactivate complement, then 1:20 diluted, followed by a 4-fold serial dilution in DMEM supplemented with 2.5% FBS, 50 units/mL penicillin–streptomycin, with each serum sample then being incubated with 150 plaque-forming units of virus (PFU) at 37°C for 1 h. The virus-serum mixtures were added onto pre-formed Vero E6 cell monolayers and incubated at 37 °C for 1 h in a 5% CO_2_ incubator. Following this, the supernatant was removed, and the cell monolayers were covered with methylcellulose overlay (final concentration: DMEM with 0.9% methylcellulose, 2% FBS and 50 U/mL penicillin–streptomycin). After 96 h, the plates were fixed with an equal volume of 8% (Servicebio, Wuhan, China) paraformaldehyde for at least 1 h, with the cell monolayers being stained with 1% crystal violet, before the plaques being counted and photographed. Neutralizing antibody titers were defined as the highest serum dilution that resulted in 50% (PRNT_50_) in the number of virus plaques reduction. The PRNT_50_ was given a value of 10 when no neutralization was observed. There were 8 serum samples at post-prime, post-initial boost and post-second boost these 3 time points while there were only 5 serum samples after challenge.

### 2.9 IFN-γ ELISpot assay

Mice spleens were collected separately and ground on a 70 μm cell sieve rinsed with mouse lymphocyte separation medium (DAKEWE, Shenzhen, China). Single cell suspensions were subsequently collected in 15 mL centrifuge tubes, with 1 mL RPMI 1640 medium (Gibco, New York, NY, USA) containing 10% FBS (R10) being added. The samples were then centrifuged at 800 × *g* at RT for 30 min. Next, the lymphocyte layer was aspirated, before adding 10 mL R10 and washing upside down. This was then centrifuged at 250 × *g* at RT for 10 min before collecting the cells. Subsequently, cells were resuspended with R10 and then counted. ELISpot assays were performed using the Mouse IFN-γ ELISpotPLUS kit (MABTECH, Nacka, Sweden) in accordance with the manufacturer’s instructions. Subsequently, 400,000 splenocytes were plated into each well and stimulated for 30 h with A29L, M1R, A35R, and B6R, at 20 μg/mL each. R10 and concanavalin A (MedChem Express, Monmouth Junction, NJ, USA) were used for negative and positive controls respectively. Spots were scanned and quantified by ImmunoSpot CTL reader (Cellular Technology Limited, Cleveland, OH, USA), and spot-forming unit (SFU) per million cells was calculated by subtracting the negative control wells. There were 3 samples from each group, and three replicates were set up for each sample.

### 2.10 Quantitative real time PCR for the detection of MPXV

Whenever commercial kits were used, the manufacturer’s instructions were followed without modification. Each tissue sample was weighed to 50 mg and then homogenized in 1 mL PBS (pH 7.4). The homogenized samples were subsequently centrifuged at 5,000 × *g* at 4°C for 10 min. Thereafter, 200 μL of the supernatant from the centrifugation was used to extract viral DNA using the DAAN Nucleic Acid Extraction or Purification Kit (DAAN Gene, Guangzhou, China). Furthermore, qPCR was performed using QuantStudio 5 real-time PCR system (Thermo Fisher Scientific, Waltham, MA, USA) and MPXV nucleic acid detection kit targeting the F3L fragment (DAAN gene, Guangzhou, China). There were 5 samples from each group, and three replicates were set up for each sample.

### 2.11 Histopathological analysis

Tissues were fixed in 4% paraformaldehyde (Servicebio, Wuhan, China) and then embedded in paraffin, followed by staining with hematoxylin and eosin (Leica, Wetzlar, Germany). Stained slides were subsequently analyzed by veterinary pathologists who were blinded to study groups.

### 2.12 Statistical analysis

All statistical analyses were performed using GraphPad Prism 8 software (GraphPad Software, San Diego, CA, USA). Unpaired Student’s t-tests were used to compare these data, with significance being set at p < 0.05.

## 3 Results

### 3.1 Characterization of multiple recombinant MPXV vaccines from outer membrane proteins constructs and immunization in mice

**Figure 1:**
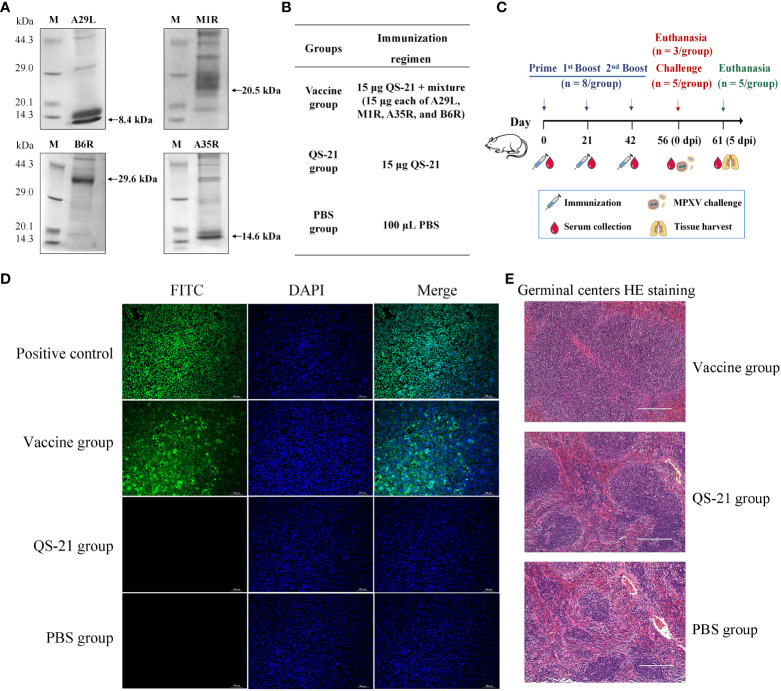
Characterization of multiple recombinant MPXV vaccines from outer membrane proteins constructs and immunization in mice. **(A)** Detection of MPXV A29L, A35R, M1R, and B6R proteins using western blot. **(B)** Experimental groups and immunisation regimens. **(C)** Schematic diagram of immunization regimens. Mice were injected subcutaneously on days 0, 21 and 42, n=8/group. Two weeks after the third immunization, mice were inoculated intranasally and intraperitoneally with MPXV, n=5/group. **(D)** Immunofluorescence staining with mouse serum to verify the expression of binding antibody. The primary antibody for the positive control was 6x His Tag Monoclonal Antibody, and for the rest of the group, mouse serum was collected after the second boost (D56). **(E)** HE staining of spleen sections from mice after the second boost (D56) to observe enlargement of the germinal centers induced by the immune response to vaccination.

MPXV proteins A29L, M1R, A35R, and B6R were measured using Western blot analysis, which revealed bands approximate to the predicted protein molecular weights of 8.4 kDa, 20.5 kDa, 14.6 kDa, and 29.6 kDa respectively (**Figure 1A**). The mice were vaccinated three times and challenged with MPXV on day 56, then animals were euthanasia and samples were collected 5 days later (**Figures 1B, C**). As a result of immunofluorescence studies, Vero E6 cells transfected with the His-tag can be detected with the mice sera from the vaccine group, whereas neither the QS-21 group, nor the PBS group. (**Figure 1D**). Spleen sections from mice in the vaccine group after immunization also showed a larger area of germinal centers observed compared to the QS-21 and PBS group (**Figure 1E**), indicating enhanced immune response.

### 3.2 Multiple recombinant MPXV vaccines induced high-titer antibody responses

**Figure 2:**
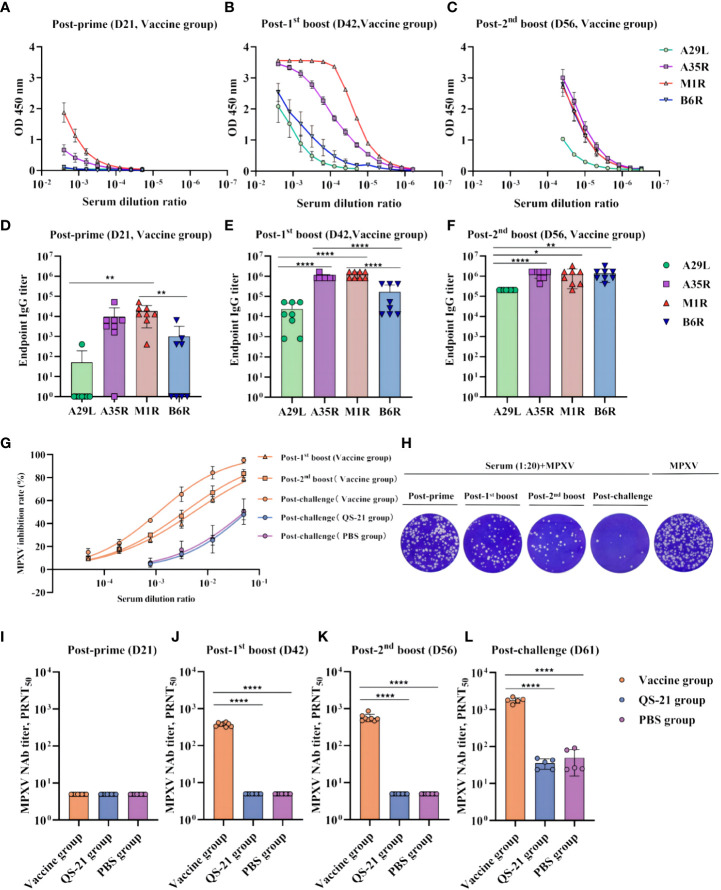
Humoral immune responses in multiple recombinant vaccine immunization in mice. **(A–C)** Measurement of serum on the day of post-prime, post-initial boost, post-second boost, and post-challenge (D21, D42, D56, and D61) binding antibodies against MPXV A29L, A35R, M1R, and B6R proteins by ELISA. Data shown represent mean OD 450 nm values (mean + SD) for each group of eight mice, n = 8/group. **(D–F)** Endpoint IgG titers by ELISA, n = 8/group. **(G)** Inhibition rate of MPXV virus by neutralizing antibodies in serum as measured by PRNT, n = 8/group at post-prime, post-initial boost, post-second boost, n = 5/group at post-challenge. **(H)** Plaque reduction assay for MPXV by serum at a dilution of 1:20. **(I–L)** PRNT of neutralizing antibodies against MPXV live viruses on the day of post-prime, post-initial boost, post-second boost, and post-challenge (D21, D42, D56, and D61). Data shown are geometric mean titers and are mean plus SD. Data were analyzed by one-way ANOVA with a multiple comparison test, n = 8/group at post-prime, post-initial boost, post-second boost, n = 5/group at post-challenge. P < 0.05 was considered statistically significant. *P < 0.05, **P < 0.01, ***P < 0.001, ****P < 0.0001.

To evaluate the immunogenicity of multiple recombinant MPXV vaccines, mice were inoculated subcutaneously with each protein in QS-21 adjuvant at 3-week intervals. The endpoint ELISA titers of the sera were also determined using plates coated with individual recombinant proteins. After the prime immunization, binding antibodies induced by A29L and B6R were either undetectable or minimal, whereas those induced by A35R and M1R were relatively high, yet all four proteins produced low levels of binding antibodies (**Figures 2A, D**). After the initial boost, antibodies rose sharply, although the antibodies induced by A35R and M1R were still significantly higher than those from A29L and B6R (**Figures 2B, E**). After the second boost, A35R and M1R-induced antibody levels did not change nearly, whereas the B6R-induced antibodies continued to increase, reaching the same levels as the A35R and M1R. Meanwhile, the A29L-induced antibody level also continued to rise, although it remained lower than the other three (**Figures 2C, F**). The immunogenicity of A35R and M1R was also superior to A29L and B6R, considering the higher levels of binding antibodies at each stage of immunization.

The level of neutralizing antibodies was assessed by PRNT assay with live MPXV in the serum at each stage after vaccination, which represented the protective effect of the vaccine against MPXV virus in mice (**Figures 2G, H**). The results showed that neutralizing antibody levels were so low as to be undetectable after prime immunization (**Figure 2I**). However, after the first boost, neutralizing antibody titers of all eight vaccinated mice reached 300 (**Figure 2J**). Furthermore, after the second boost, the geometric mean of neutralizing antibody titer in the eight mice was 574, with the highest being 865 (**Figure 2K**). Notably, after virus challenge, there was a significant neutralizing antibody increase in the vaccine group, with all five mice exceeding 1300 and the highest being 2188. Neutralizing antibodies were also detected in the QS-21 and PBS groups, representing a normal functioning immune system in all mice, the virus stimulated mice to produce neutralizing antibodies, but the antibody titer did not exceed 100 (**Figure 2L**).

### 3.3 Immunization of mice with multiple recombinant MPXV vaccines induced strong cellular immune responses

**Figure 3:**
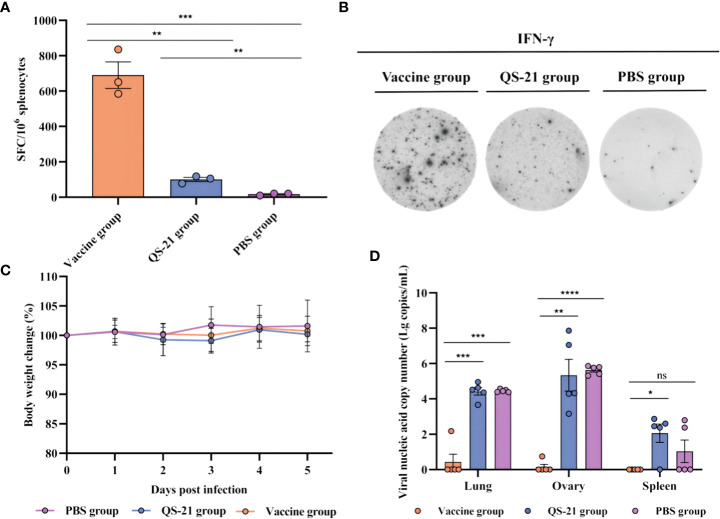
Cellular immune responses and protection against challenges of MPXV in multiple recombinant vaccine immunization in mice. **(A, B)** T cell responses against MPXV proteins A29L, M1R, A35R, and B6R as measured by IFN-γ ELISpot. Three mice from each group were euthanized 2 weeks after the second boost, before splenocytes were harvested, and a single-cell suspension was stimulated for 30 h with recombinant proteins of A29L, M1R, A35R, and B6R, n = 3/group, each sample contained three replicates. **(C)** Body weight changes following virus challenges, n = 5/group. **(D)** qPCR detection of viral loads in the lungs, ovaries, and spleen. Data shown are mean plus SD. Data were analyzed by one-way ANOVA with a multiple comparison test. P < 0.05 was considered statistically significant, n = 5/group, each sample contained three replicates. * P < 0.05, ** P < 0.01, *** P < 0.001, **** P < 0.0001.

T cell responses against MPXV proteins A29L, M1R, A35R, and B6R were detected by IFN-γ ELISpot. Three mice from each group were euthanized 2 weeks after receiving the second boost, and the splenocytes were harvested. A single-cell suspension was stimulated for 30 h with recombinant proteins of A29L, M1R, A35R, and B6R. As expected, the vaccine induced significantly higher levels of IFN-γ + T cells compared to the QS-21 and PBS groups on day 56 (**Figures 3A, B**). Average 690 spots in the vaccine group, 101 spots in the QS-21 group, and 17 spots in the PBS group.

### 3.4 Multiple recombinant MPXV vaccines significantly inhibited virus proliferation

Some literature states that MPXV multiplies in BALB/c mice and peaks on days 4–7, and the highest viral loads were observed in the ovaries and lungs, followed by progressive clearance, and viral challenge had no major effect on body weight or the mental status of the mice (28, 29, 31). We therefore chose to euthanize mice on day 5 of virus infection. Body weight data from our experiments showed the same trend, with no significant differences between any of the three groups of mice (**Figure 3C**). Additionally, qPCR results showed significant differences in viral load within the lungs, ovaries, and spleens of the vaccine group mice compared to the QS-21 group, as well as in the lungs and ovaries of the vaccine group mice compared to the PBS group. The highest amount of virus detected in the tissues of mice in the vaccine group was 10^2.2^ copies/mL, while the highest amount of virus detected in the tissues of mice in the QS-21 group reached 10^7.9^ copies/mL for the ovaries. The highest viral load in the PBS group was also in the ovaries, reached 10^5.9^ copies/mL (**Figure 3D**). The results of the attack-protection assay sufficiently demonstrate the ability of our vaccine to inhibit the multiplication of the virus in mice.

### 3.5 Histopathological changes in mice infected with MPXV

**Figure 4:**
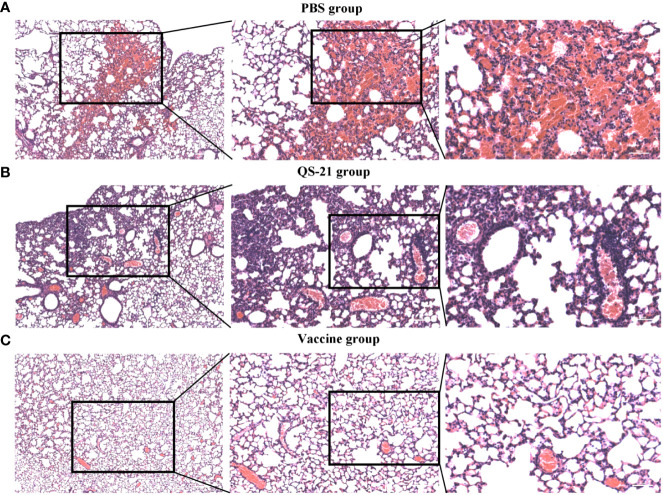
HE staining of lung sections from mice after virus challenge to visualize lung lesions. **(A)** Lung sections of mice in PBS group. **(B)** Lung sections of mice in QS-21 group. **(C)** Lung sections of mice in vaccine group. Scale bars from left to right are 200 μm, 100 μm, 50 μm.

All mice showed histopathological changes in the lungs on day 5 after the challenge, including five mice in the vaccine group, thus indicating that virus infection had occurred. However, the extent of the lesions in the vaccine group was not as severe as observed in the control group. Mice in the PBS and QS-21 groups showed extensive alveolar cell necrosis with significant congestion in the necrotic alveoli, alongside a large infiltration of lymphocytes and neutrophils. Additionally, some of the alveolar walls were thickened, congested, and edematous. Additionally, the alveolar septum was significantly widened, and the blood vessels were dilated and congested and interstitial was infiltrated by a large number of inflammatory cells. In contrast, the histopathological changes in the lungs of the mice in the vaccine group were generally dilated and congested lung vessels, with a small amount of inflammatory cell infiltration in the interstitial and slight thickening of the alveolar walls (**Figure 4**).

## 4 Discussion

Following the occurrence of the 2022 mpox epidemic, attention quickly shifted to the development of a mpox vaccine based on the lessons learned from the COVID-19 outbreak. Although previous studies have shown that smallpox vaccines offer some protection against both MPXV and current mutant strains, this also raised similar concerns around the safety of an attenuated smallpox virus vaccine. In contrast, the WHO has declared the smallpox virus to be extinct and it is less feasible to reproduce an attenuated smallpox vaccine. According to Madeley CR, inactivated vaccinia virus vaccine does not produce satisfactory immunity (32). Inactivated mpox vaccines are unable to stimulate sufficient immunity in the body, since there are many important neutralizing epitopes on the EEV particles of MPXV, and inactivation of the virus would lose these important antigens. Protein subunit vaccines have an excellent safety profile and can provide good protection when combined with adjuvants for immunization. Another important point is that the 2022 mpox epidemic was concentrated within the homosexual community, which is also vulnerable to a higher prevalence of AIDS (33–38). The immunodeficiency caused by AIDS can prevent them from being immunized with live attenuated vaccines, which provides a powerful shock to their immune system (39–42), and therefore a recombinant protein vaccine may be a better option for them. In this study, we demonstrate that multiple recombinant vaccines can trigger antibodies against both IMV and EEV forms of MPXV. Three immunizations of mice with a recombinant protein-linked QS-21 adjuvant activated cellular and humoral immunities and induced high titers of binding antibodies, however, against each protein at different titers. More importantly, the neutralizing antibodies induced by this vaccine sufficiently inhibited the multiplication of the virus, while protecting the mice from persistent infection by MPXV. In addition, the level of antibodies correlates with the number of immunizations and this result may further guide the designation of immunization strategies in the future.

IFN-γ plays an important role in the induction of antiviral immunity as a cytokine secreted by immunoreactive cells. Once Th1 cells have matured, they secrete IFN-γ and promote more Th1 cell differentiation. IFN-γ secreted by Th1 cells activates macrophages, while also stimulating B cells to produce receptors that enhance viral attachment to macrophages (43). IFN is one of the primary innate mediators after viral infection, and susceptibility to and severity of the infection are both increased when IFN is insufficient (44). The ability of mouse splenocytes to secrete IFN-γ was significantly stronger within the vaccine group than in the control group, as measured by ELISpot, thus indicating that the vaccine elicited a strong T-cell immune response, as indicated by the increased size and number of spleen germinal centers in mice. Although protein vaccines generally require multiple immunizations, the high titer of binding antibodies produced by three immunizations of A29L, M1R, A35R, and B6R mixed with QS-21 demonstrated good immunogenicity of this protein vaccine. After the second boost, the geometric mean of neutralizing antibody titer in the eight mice was 574, with the highest being 865. An article published in November 2022 on the MPXV mRNA vaccine also chose BALB/c mice as experimental animals and the encoded proteins chosen were A35R and M1R, which were immunized twice on days 0 and 14, and serum was collected on day 35, and both vaccine groups (VGPox 1 and VGPox 2) showed over 80% inhibition at a dilution of 1:500 (45). Another MPXV DNA vaccine based on vaccinia virus genes (L1R, A27L, A33R, and B5R) produced a mean titer of 9600 conjugated antibodies and a mean titer of 373 neutralizing antibodies in an monkey model (46). And there are just as many reviews of attenuated vaccines. Hatch GJ (47) compared ACAM-2000, a second-generation smallpox vaccine manufactured by Dryvax/NYCBH, with Imvamune, a third-generation smallpox vaccine manufactured by Bavarian Nordic, on a crab-eating monkey model. After vaccination with ACAM-2000, the maximum median titer at 6 days before challenge was 132 U/ml animals receiving two Imvamune boosters reached 69 U/ml at 6 days before challenge, which was significantly higher than the titer in the single-dose Imvamune group (P < 0.05). In the MPXV challenge, all monkeys immunized with ACAM-2000 and twice with Imvamune survived, all control monkeys died, while two monkeys died in the group immunized with only one dose of Imvamune. Another article directly compares the efficacies of MVA alone and in combination with classical VV-based vaccines in a cynomolgus macaque mpox model. Four vaccine strains, MVA-BN, IMVAMUNE, Elstree-RIVM and Elstree-BN, were included. However, the neutralization titers at 9 weeks after last vaccination were low, with MVA-BN, IMVAMUNE and Elstree-BN not exceeding 100 (48). Although attenuated vaccines do not show a significant advantage in stimulating the production of neutralizing antibodies in the organism, this may be due to large differences in animal models and evaluation methods between studies that do not facilitate comparison. One aspect of note is that Several studies have shown that antibodies persist in the host for several years after attenuated smallpox vaccination (49–51) and this will be an important part of future evaluations of subunit vaccines such as recombinant protein and nucleic acid vaccines. Furthermore, it is interesting to note that according to our results, the titer of binding antibodies against A29L was consistently the lowest; According to Hooper JW, vaccination with A27L (homologous to mpox virus A29L) alone failed to protect mice from vaccinia virus (52). Future experiments should be designed aiming to verify whether A29L plays a key role in this vaccine.

We believe that the antibody targeting strategy for the MPXV contributed significantly to the protection of protein-immune mice from infection. Antibodies produced by immunized mice prevented virus recognition, entry into host cells, and the spread of mature viruses between cells. Protein immunization alone does not completely protect mice from a viral infection, since the antibodies they produce may only act on one stage of viral infection. *In vitro* testing of immune sera to neutralize IMV showed that a combination of A29L-, M1R-, A35R-, and B6R-specific antibodies was very effective in inactivating the MPXV. This was also supported by the results of the mpox virus attack challenge. Furthermore, our results are consistent with those published by Osorio JE (29), whereby MPXV was replicated heavily in the ovaries, lungs, and spleens of mice, and high viral loads were detected in the tissues of control mice and were almost undetectable in those of vaccinated mice. We believe that neutralizing antibodies in vaccine group mice protect them from MPXV challenge. Notably, the serum levels of neutralizing antibodies in the vaccine group of mice that received the virus challenge were substantially elevated, with an average antibody titer of 1785 in the five mice. The neutralizing antibody titer after the second boost was 574, with an average threefold increase observed within 5 days of the virus challenge, again indicating that the three prior immunizations had established a complete immune system response in the mice against MPXV. Therefore, antibody titers increased 3-fold upon exposure to a large number of viral attacks, and this increase occurred even after several neutralizing antibodies had bound to the antigen and been cleared together. Although this vaccine does not prevent the virus from causing histopathological changes in the lung, it induces antibodies that neutralize the virus and moderate tissue damage from the virus by reducing the virus titer and area of infection. This reinforced the fact that the vaccine provided complete protection in mice from MPXV persistent infection.

A combination of these four proteins and QS-21 provided complete protection, although which specific protein played the key role in immune protection was not investigated in this study, thus more research is required to explore the specificity of the antibody response. However, depending on the ELISA results, the immunization dose of each protein could be optimized to achieve optimal immunization. In terms of the cellular immune response, we were unable to schedule ELISpot assay for the four proteins stimulated individually to further evaluate the individual effect of each protein in the ability of immune cells to produce IFN-γ, considering the insufficient total number of spleen cells. All of the above await our further adjustment and evaluation of this vaccine in the future. In conclusion, this study demonstrated the feasibility of a multiple recombinant MPXV vaccination with A29L, M1R, A35R, and B6R.

## Data availability statement

The original contributions presented in the study are included in the article/**Supplementary Material**. Further inquiries can be directed to the corresponding authors.

## Ethics statement

The animal study was reviewed and approved by the animal ethics committee of WIBP (Ethics No. WIBP-A II 442022006). Wuhan Institute of Biological Products Co., Ltd., Wuhan City, China.

## Author contributions

XGL, ZW, and JL designed and supervised the study. DT, XKL, HF, XX, KS, RW, CL, DD, and HD performed the experiments. DT, XKL and JL performed the statistical analysis. DT and XKL wrote the manuscript. XGL, ZW, and JL revised the manuscript. All authors contributed to the article and approved the submitted version.
