# Supplementary figures and images for: Recombinant proteins A29L, M1R, A35R, and B6R vaccination protects mice from mpox virus challenge
Source: Front Immunol. 2023 Jun 26;14:1203410. doi: 10.3389/fimmu.2023.1203410 (PMC10331816; doi:10.3389/fimmu.2023.1203410)

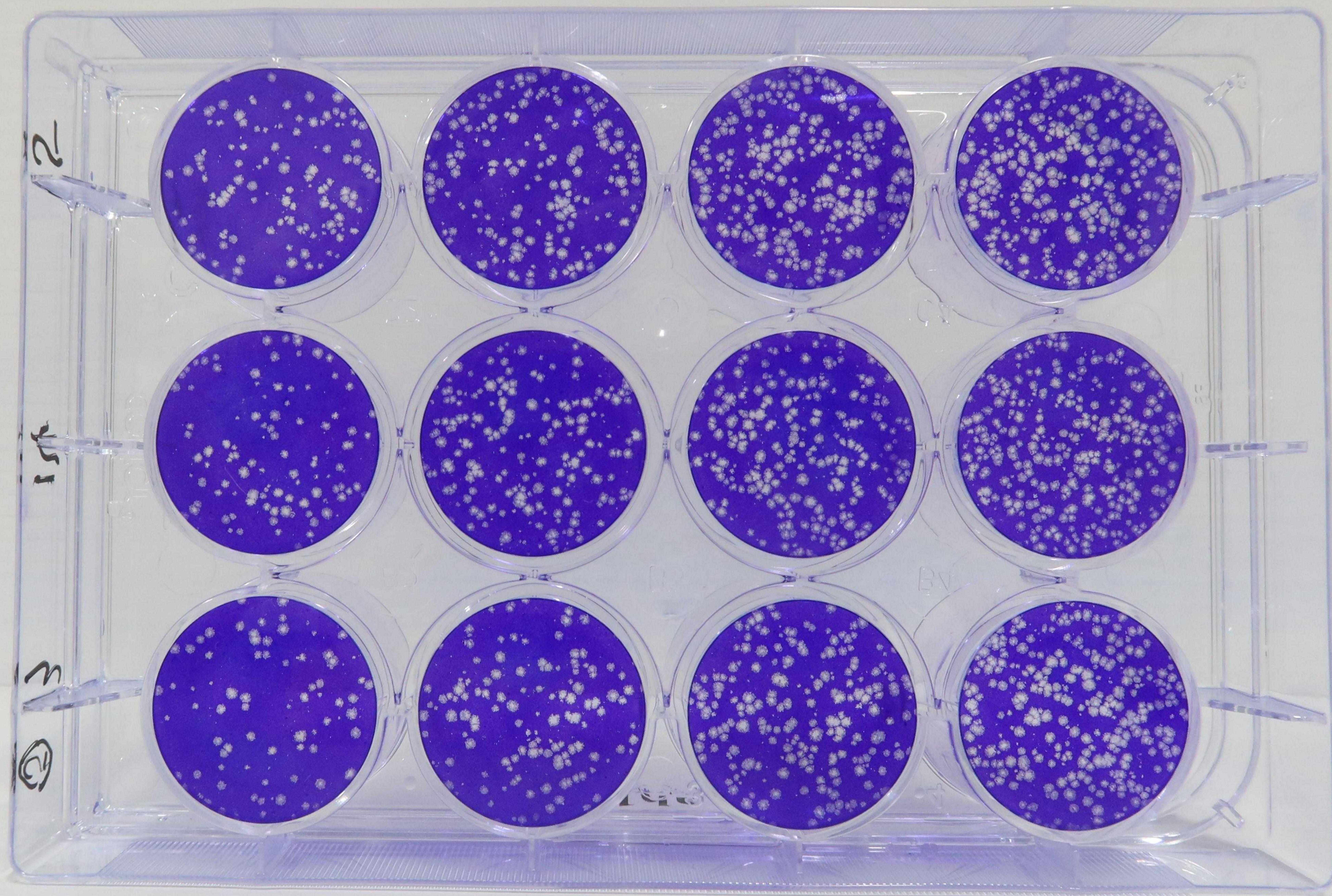

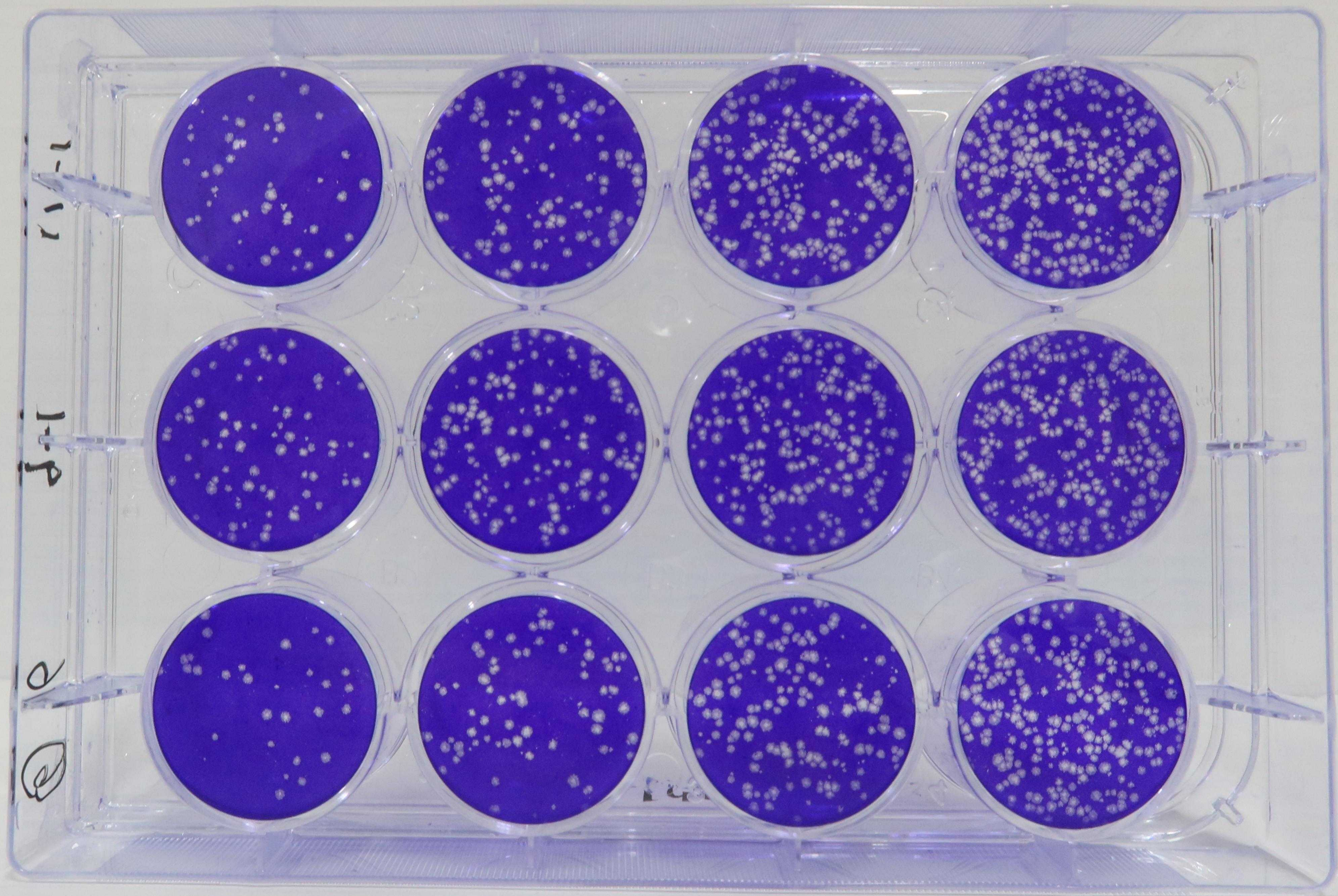

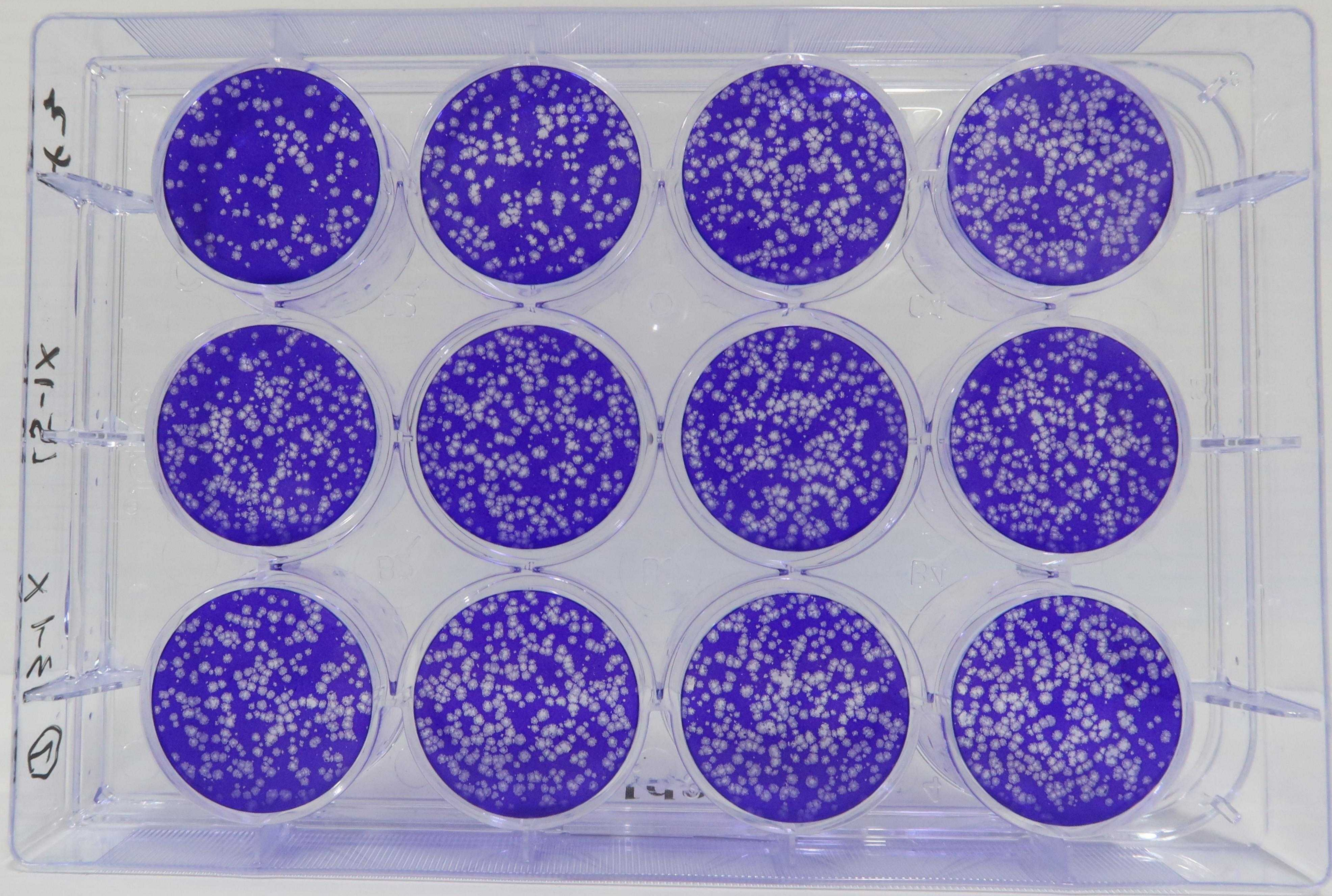

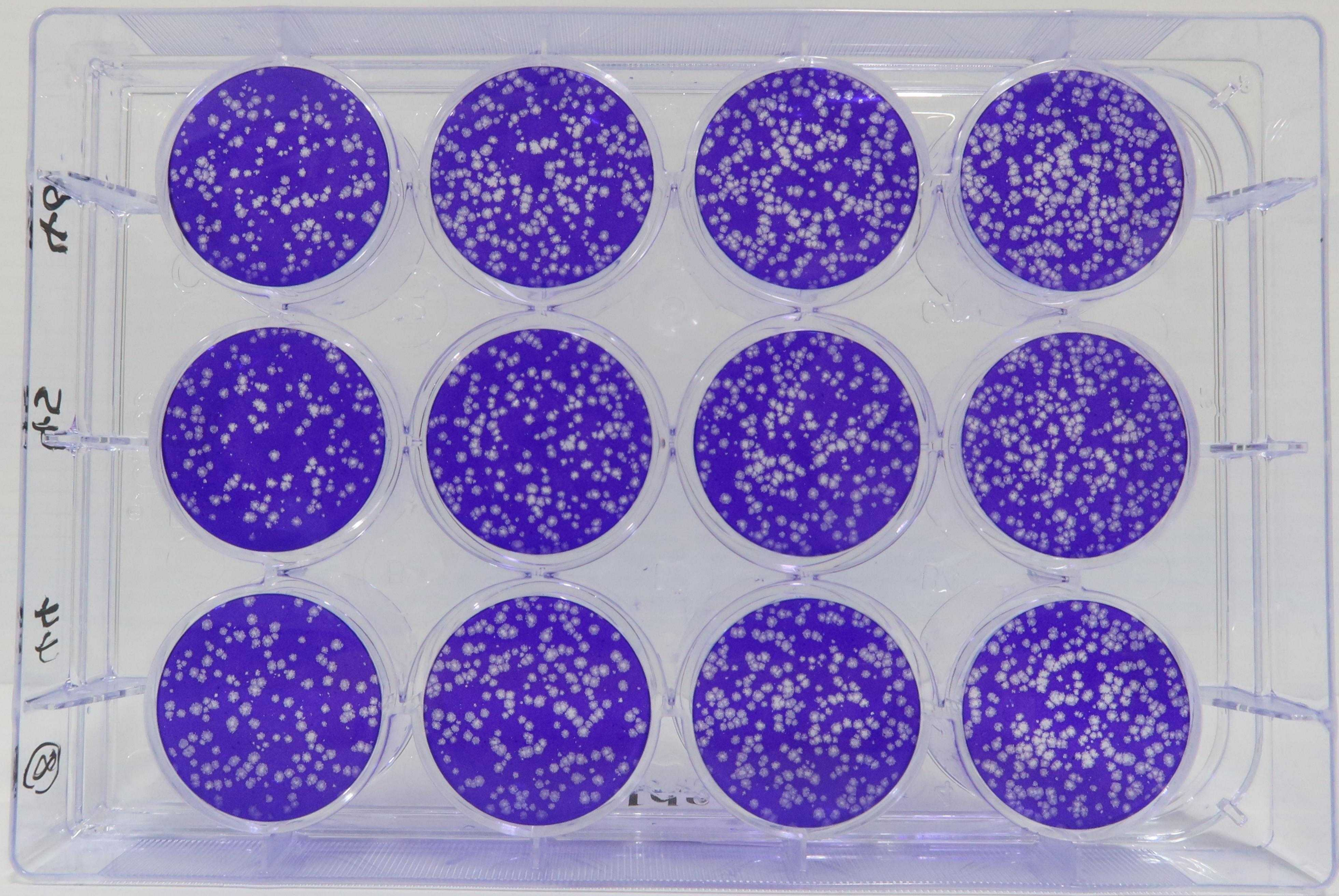

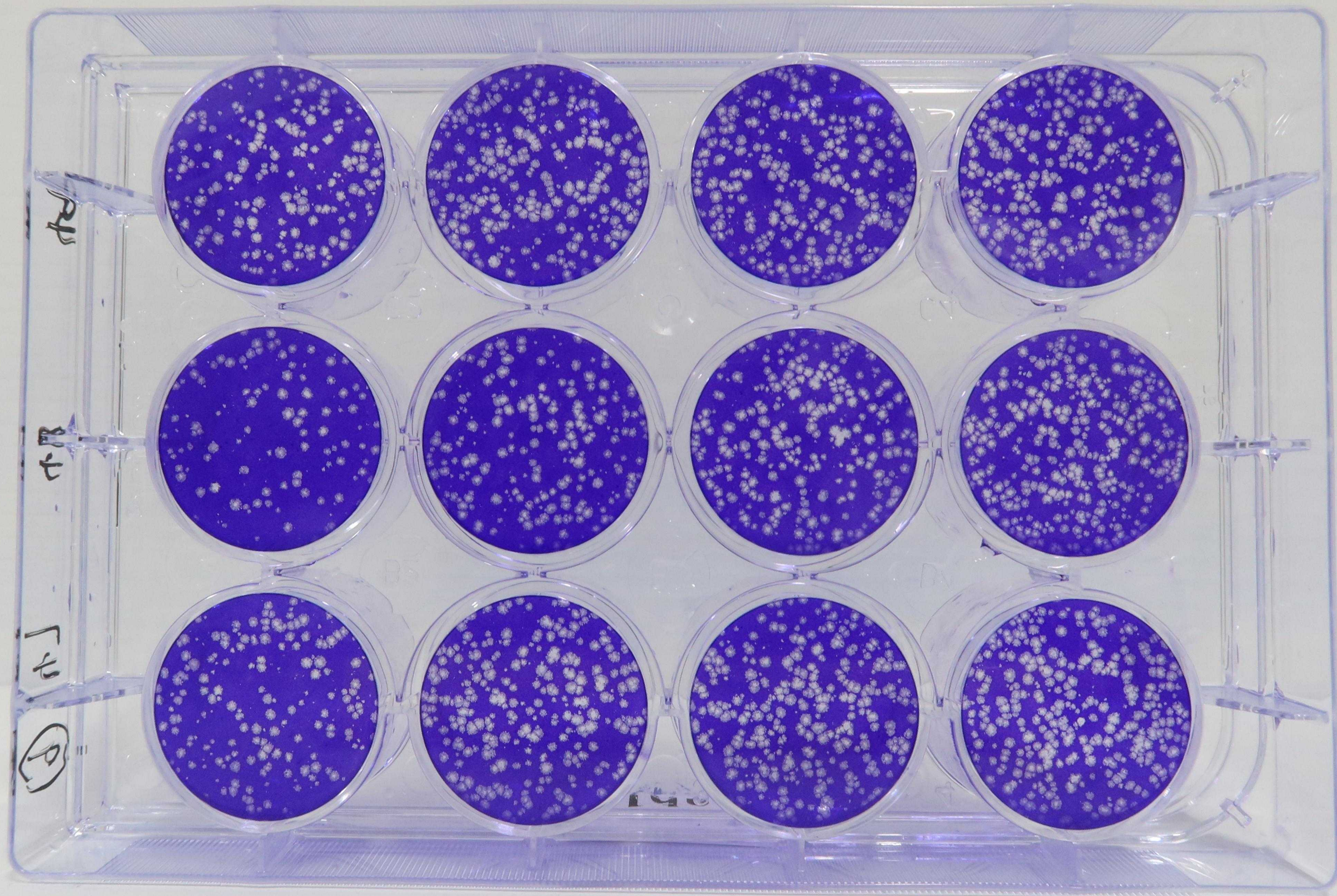

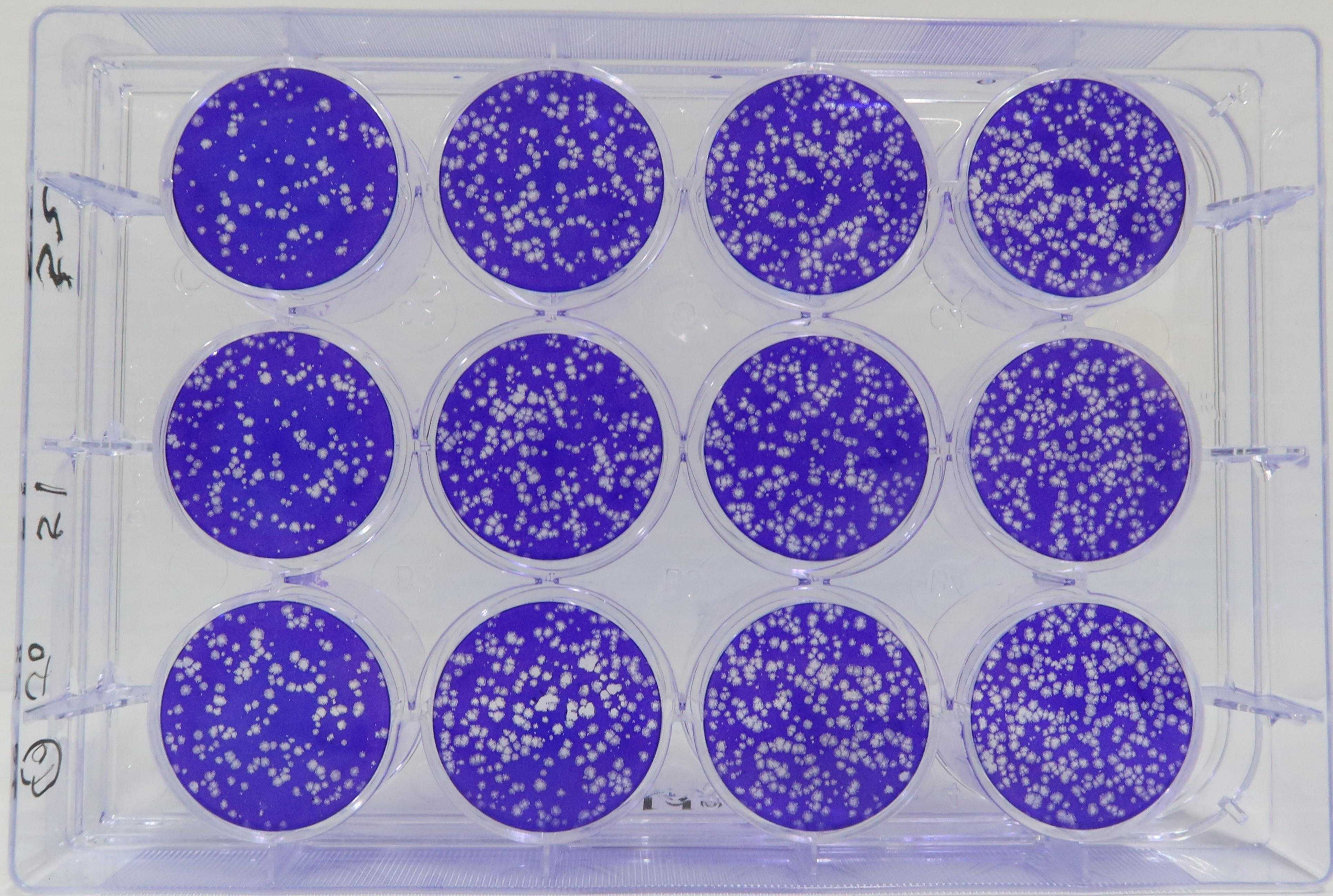

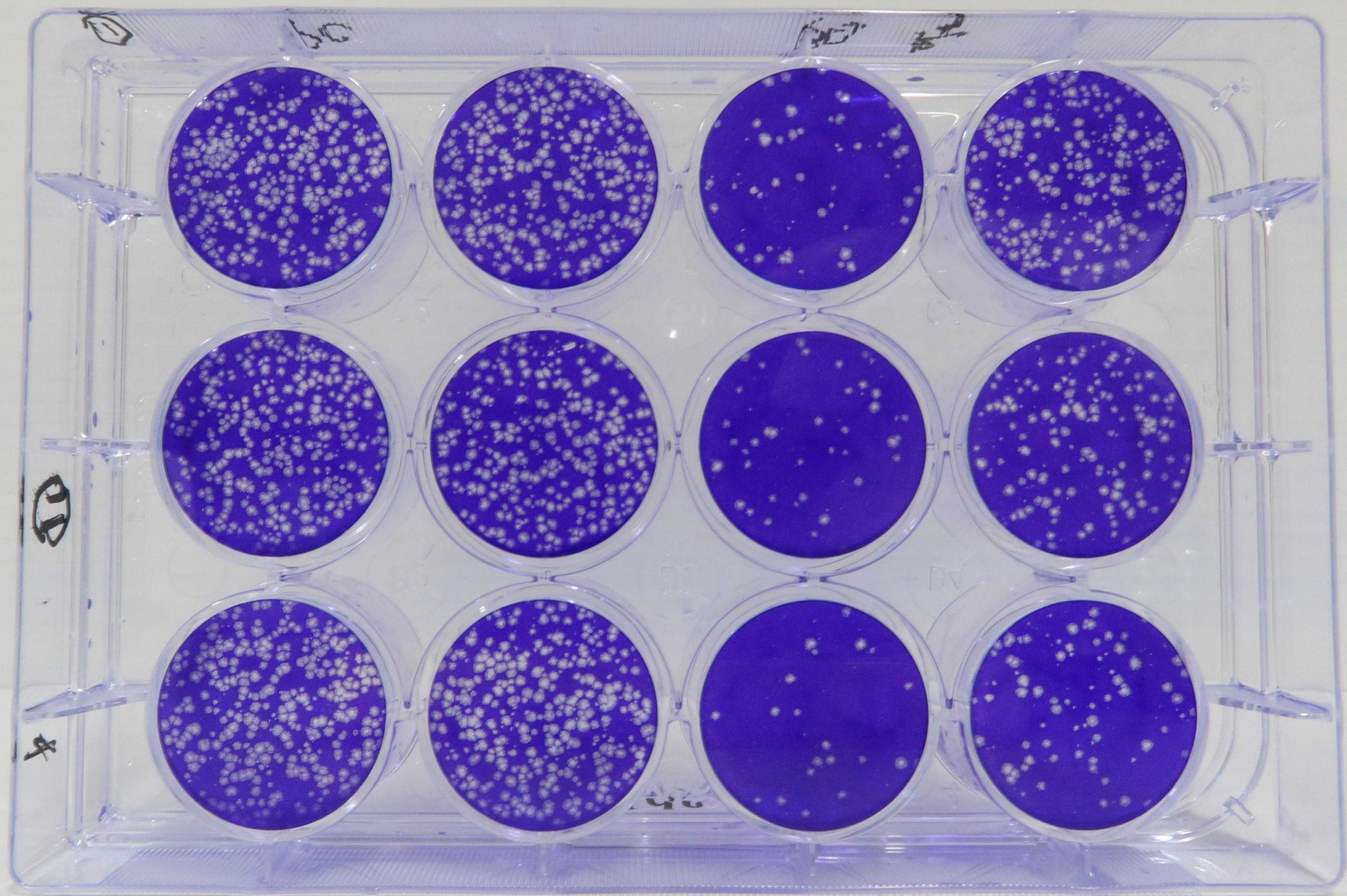

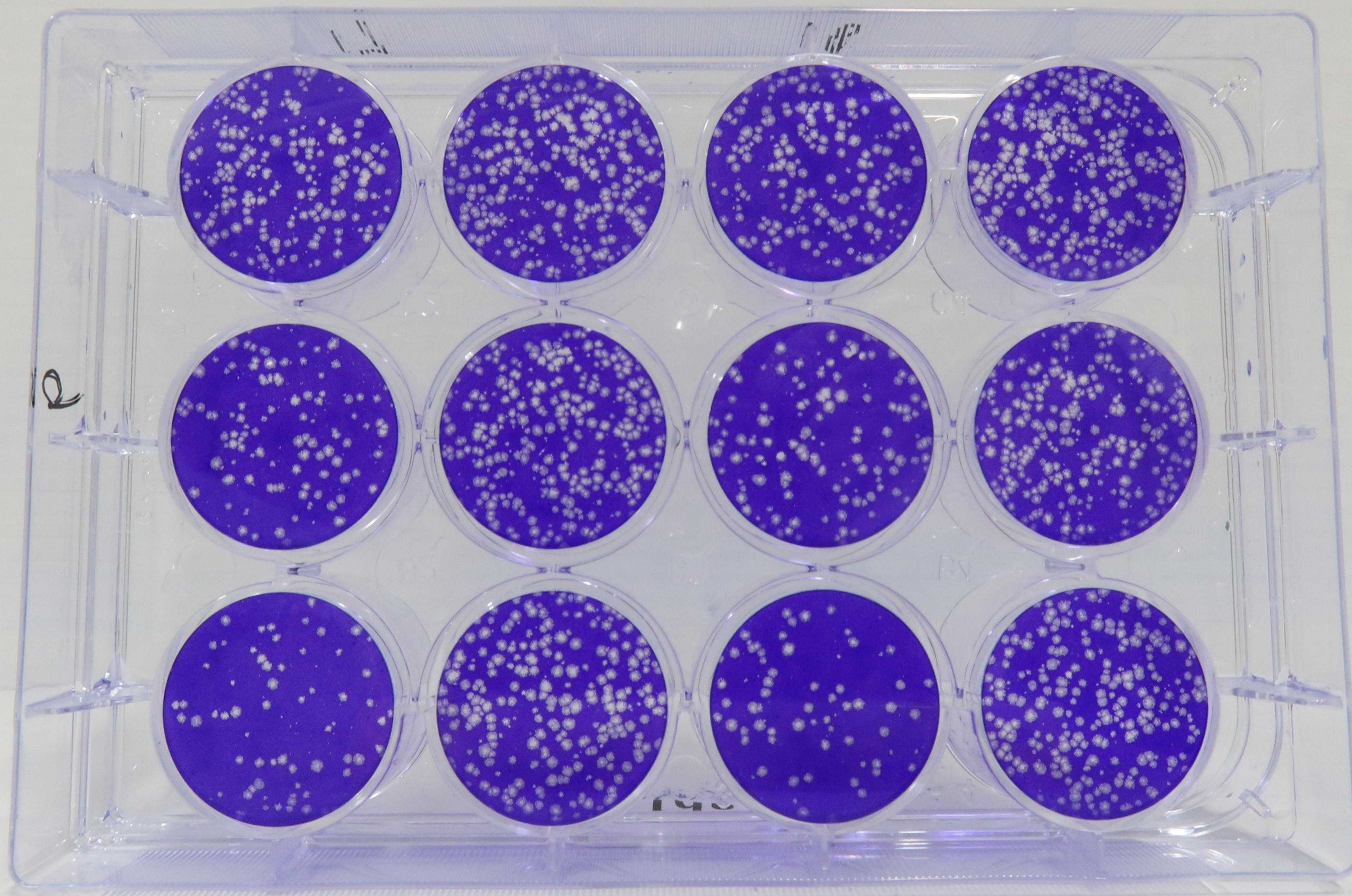



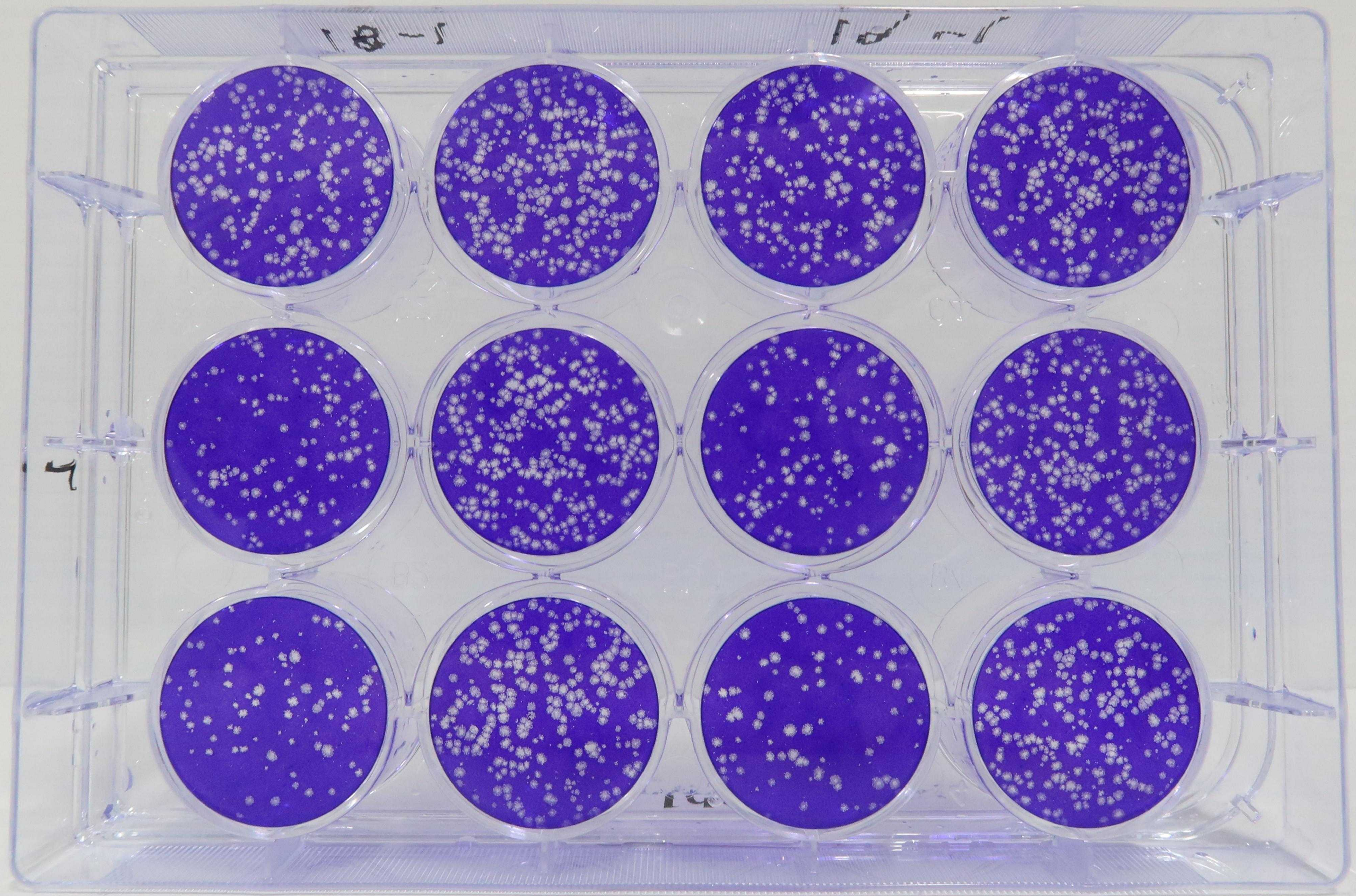

Supplement: Supplementary file 1 [file DataSheet_1.pdf]

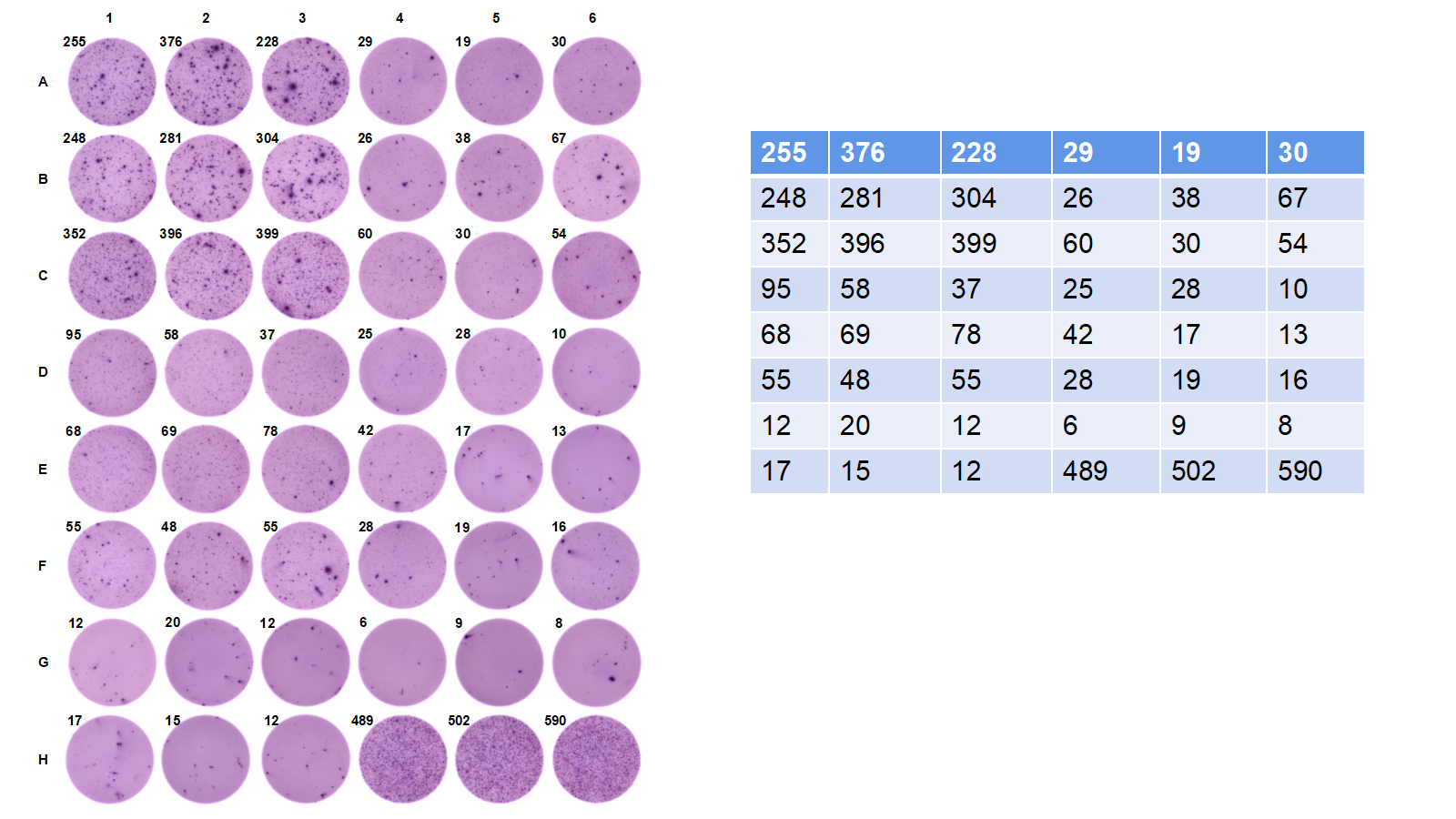

Supplement: Supplementary file 2 [file Image_1.tif]

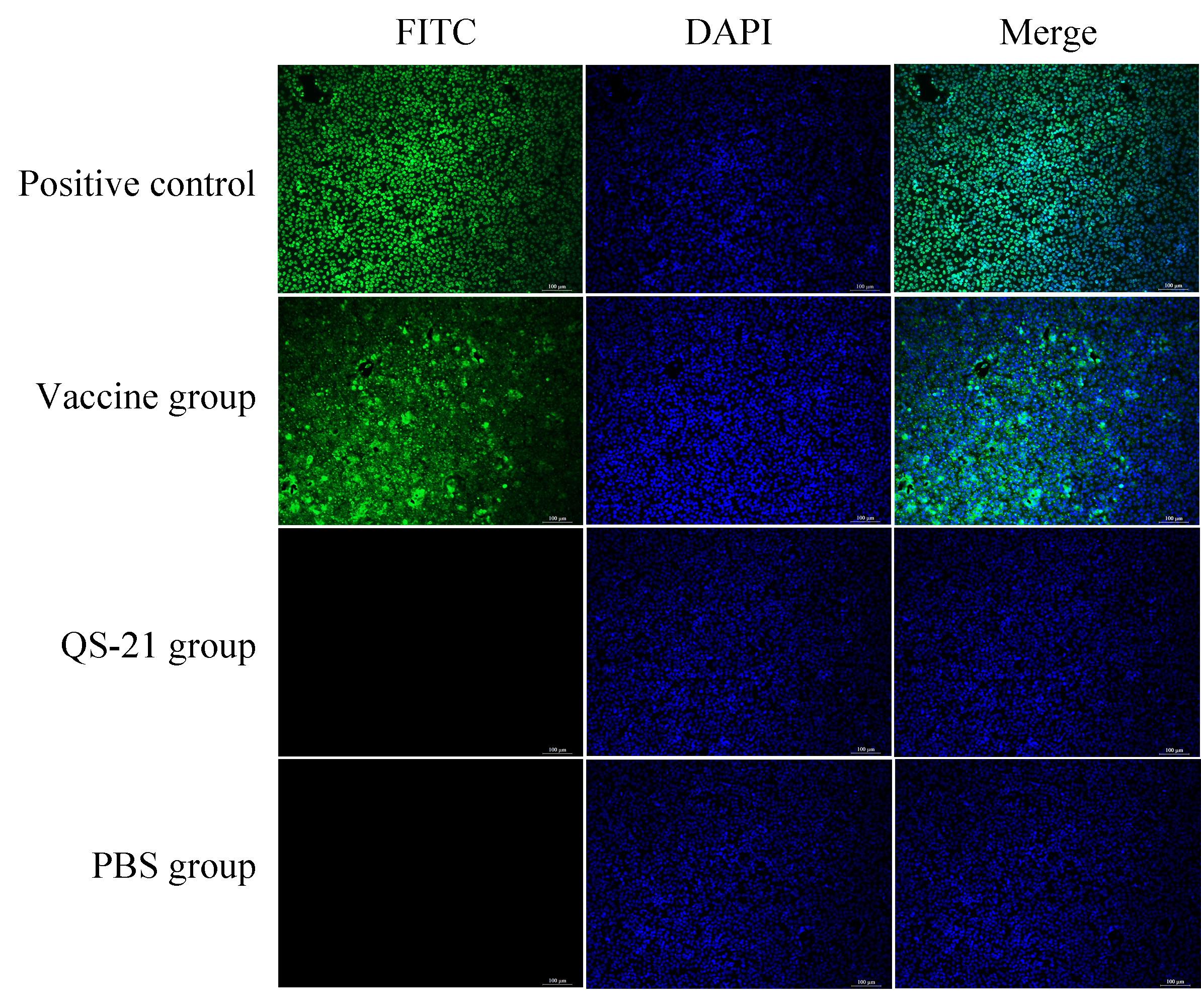

Supplement: Supplementary file 3 [file Image_2.tif]

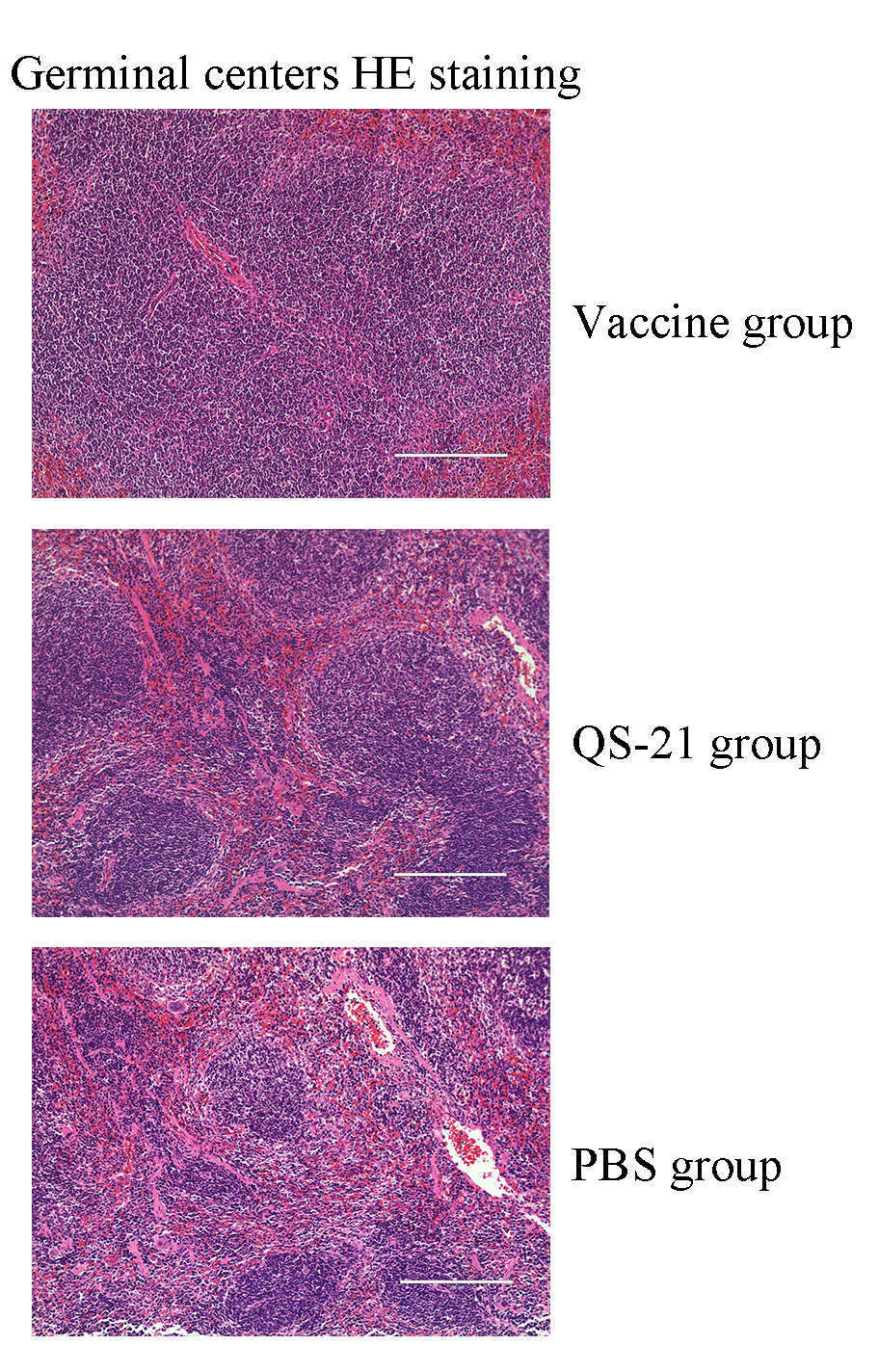

Supplement: Supplementary file 4 [file Image_3.tif]

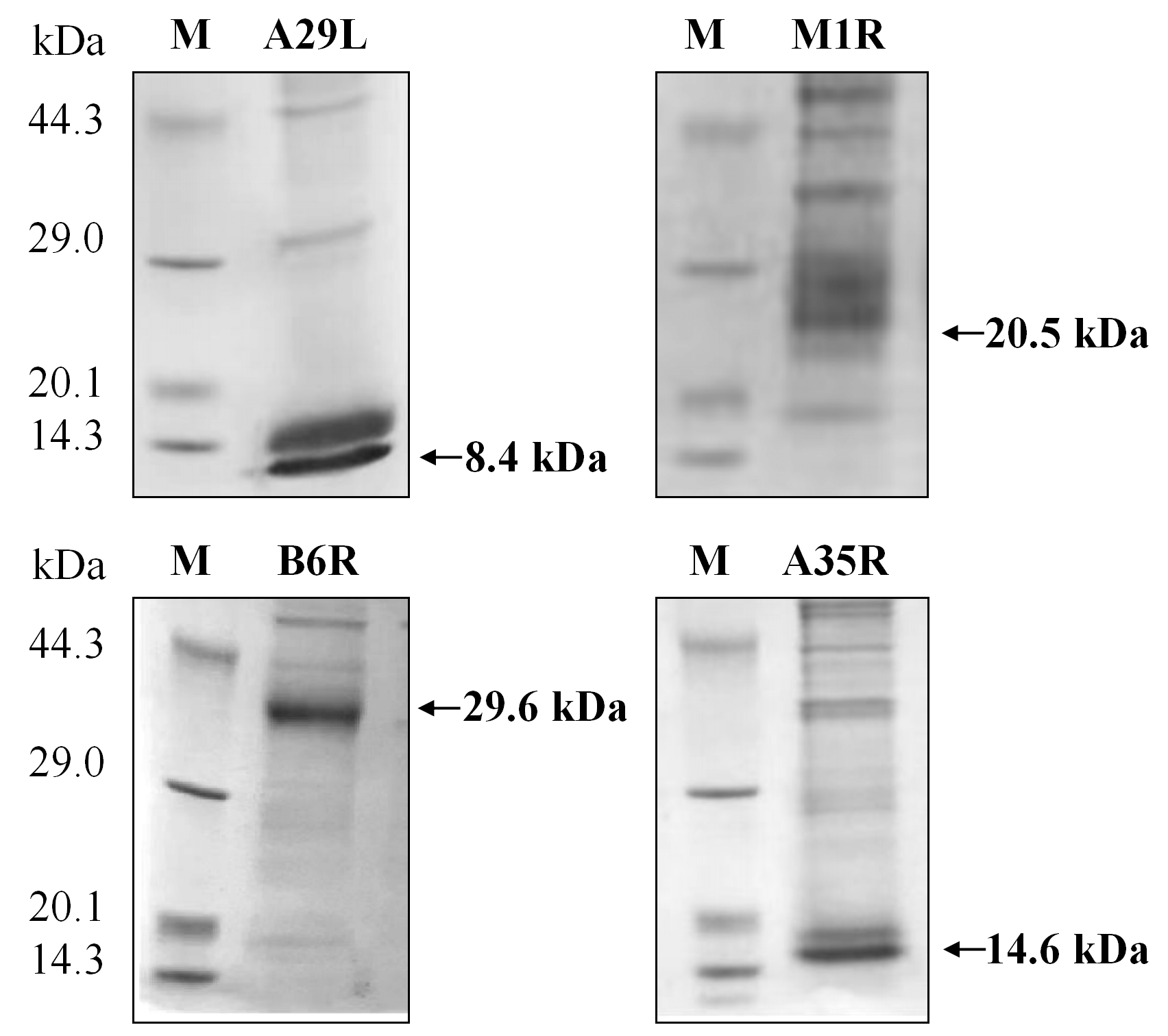

Supplement: Supplementary file 5 [file Image_4.tif]
